# BAck complaints in the elders - chiropractic (BACE-C): protocol of an international cohort study of older adults with low back pain seeking chiropractic care

**DOI:** 10.1186/s12998-020-00302-z

**Published:** 2020-04-01

**Authors:** Alan D. Jenks, Trynke Hoekstra, Iben Axén, Katie de Luca, Jonathan Field, Dave Newell, Jan Hartvigsen, Simon D. French, Bart Koes, Maurits W. van Tulder, Sidney M. Rubinstein

**Affiliations:** 1 Department of Health Sciences and Amsterdam Movement Science, Faculty of Science, Vrije Universiteit, De Boelelaan 1085, room WN U601, 1081HV, Amsterdam, The Netherlands; 2 Department of Health Sciences and Amsterdam Public Health research institute, Faculty of Science, Vrije Universiteit, Amsterdam, Netherlands; 3 Unit of Intervention and Implementation Research for Worker Health, Institute of Environmental Medicine, Karolinska Institutet, Stockholm, Sweden; 4 Department of Chiropractic, Faculty of Science and Engineering, Macquarie University, Sydney, Australia; 5 Back Active, Private Practice, Southsea, Portsmouth, UK; 6 AECC University College, Bournemouth, UK; 7 Department of Sports Science and Clinical Biomechanics, University of Southern Denmark, Odense M, Denmark; 8 Nordic Institute of Chiropractic and Clinical Biomechanics, University of Southern Denmark, Odense, Denmark; 9 Department of General Practice, Erasmus University Medical Center, Rotterdam, The Netherlands; 10 Center for Muscle and Joint Health, University of Southern Denmark, Odense, Denmark; 11 Department of Physiotherapy & Occupational Therapy, Aarhus University Hospital, Aarhus, Denmark

**Keywords:** Ageing, Chronic pain, Epidemiology, Low back pain, Aged, Elderly, Spinal manipulation, Chiropractic

## Abstract

**Background:**

Low back pain is a common condition among older adults that significantly influences physical function and participation. Compared to their younger counterparts, there is limited information available about the clinical course of low back pain in older people, in particularly those presenting for chiropractic care. Improving our understanding of this patient population and the course of their low back pain may provide input for studies researching safer and more effective care than is currently provided.

**Objectives:**

The primary objectives are to examine the clinical course over one year of pain intensity, healthcare costs and pain, functional status and recovery rates of low back pain in people 55 years and older who visit a chiropractor for a new episode of low back pain.

**Methods:**

An international prospective, multi-center cohort study with one-year follow-up. Chiropractic practices are to be recruited in the Netherlands, Sweden, United Kingdom and Australia. Treatment will be left to the discretion of the chiropractor. Inclusion/Exclusion criteria: Patients aged 55 and older who consult a chiropractor for a new episode of low back pain, meaning low back pain for the first time or those patients who have not been to a chiropractor in the previous six months. This is independent of whether they have seen another type of health care provider for the current episode. Patients who are unable to complete the web-based questionnaires because of language restrictions or those with computer literacy restrictions will be excluded as well as those with cognitive disorders. In addition, those with a suspected tumor, fracture, infection or any other potential red flag or condition considered to be a contraindication for chiropractic care will be excluded. Data will be collected using online questionnaires at baseline, and at 2 and 6 weeks and at 3, 6, 9 and 12 months.

**Discussion:**

This study, to our knowledge, is the first large-scale, prospective, multicenter, international cohort study to be conducted in a chiropractic setting to focus on older adults with low back pain consulting a chiropractor. By understanding the clinical course, satisfaction and safety of chiropractic treatment of this common debilitating condition in the aged population, this study will provide input for informing future clinical trials.

**Trial registration:**

Nederlandse Trial Registrar NL7507.

## Background

Worldwide, low back pain is the leading cause of years lived with disability and contributes to the global burden of disease [1, 2]. Low back pain is associated with decreased mobility, reduced social participation, increased isolation and difficulty with activities of daily living and thus has a negative effect on overall health-related quality-of-life in older adults. Older adults with low back pain also more commonly suffer from a range of co-morbidities when compared to those without low back pain [3, 4]. This results in large costs of care, which are estimated to exceed €400 billion per year worldwide [5].

Low back pain is generally more severe with increasing age [6]. For example, one in every four people aged > 80 years will report moderate to severe low back pain and people aged > 80 years are three times more likely to have high intensity low back pain (scores > 50, on a zero to 100 scale) than those aged 50–59 years [7]. One-fifth of older adults with low back pain report difficulties in caring for themselves at home or participating in family- and social activities [8]. Older people seeking care because of low back pain more commonly receive treatments that have been shown to be ineffective and harmful such as opioid prescription, spinal injections or surgery than younger people seeking care for low back pain [9].

Chiropractors provide a significant portion of care for patients with low back pain [10], and care from chiropractors in the younger and older population appears to be safe and effective [11–13]. Unfortunately, existing trials have typically included only younger adults with low back pain, and exclude older adults for various complicating reasons, such as comorbidity and polypharmacy [14, 15]. As a significant proportion of chiropractors treat older adults [11], it is important to understand the course and characteristics of low back pain in older adults under this care. Perhaps more importantly, chiropractic care may delay functional decline in older adults and improve self-rated health [12, 13].

In short, there is a general lack of knowledge regarding low back pain in older adults, but more importantly, data are lacking on course of low back pain for this population in a chiropractic setting [14, 16].

The current BACE-C consortium study has been modelled after the ‘BAck Complaints in Elders’ study (BACE), which is an international cohort study devoted to examining back complaints in older people in primary care [17]. The BACE-C study is set in chiropractic care. The primary objectives are to examine the clinical course over one year of the intensity, healthcare costs and improvement rates of low back pain in people 55 years and older who visit a chiropractor for a new episode of low back pain.

## Methods

Study design. This study is designed as an international, multi-center prospective cohort study. Data are to be collected from patients 55 and older with low back pain who visit a chiropractor. Follow-up measurements will be scheduled at 2 weeks, 6 weeks, 3 months, 6 months, 9 months and at one year after the first treatment. Participants are to be recruited from the private practices of chiropractors in the Netherlands, Sweden, Australia and the United Kingdom using the same recruitment strategies. The procedures and design outlined in this paper are to be followed by the participating countries and describe a common set of primary outcome measures and patient- and chiropractic factors to be measured. Care will be at the discretion of the participating chiropractors. Ethics approval will be obtained in each participating country prior to data collection.

### Participants

#### Inclusion criteria

Patients aged 55 and older who consult a chiropractor for a new episode of low back pain, meaning low back pain for the first time or those patients who have not been to a chiropractor in the previous six months. This is independent of whether they have seen another type of health care provider for the current episode. All low back complaints, with pain in the region from the thoracolumbar 12th rib junction to the first sacral vertebrae, including pelvic pain and pain referral to the leg(s) are to be included. Chiropractors who are licensed and currently work in clinical practice will be asked to participate.

#### Exclusion criteria

Patients who are unable to complete the web-based questionnaires because of language restrictions or computer literacy restrictions will be excluded as well as those with cognitive disorders. In addition, those with a suspected tumor, fracture, infection or any other potential red flag or condition considered to be a contraindication for chiropractic care will be excluded.

### Inclusion procedure

**Fig. 1:**
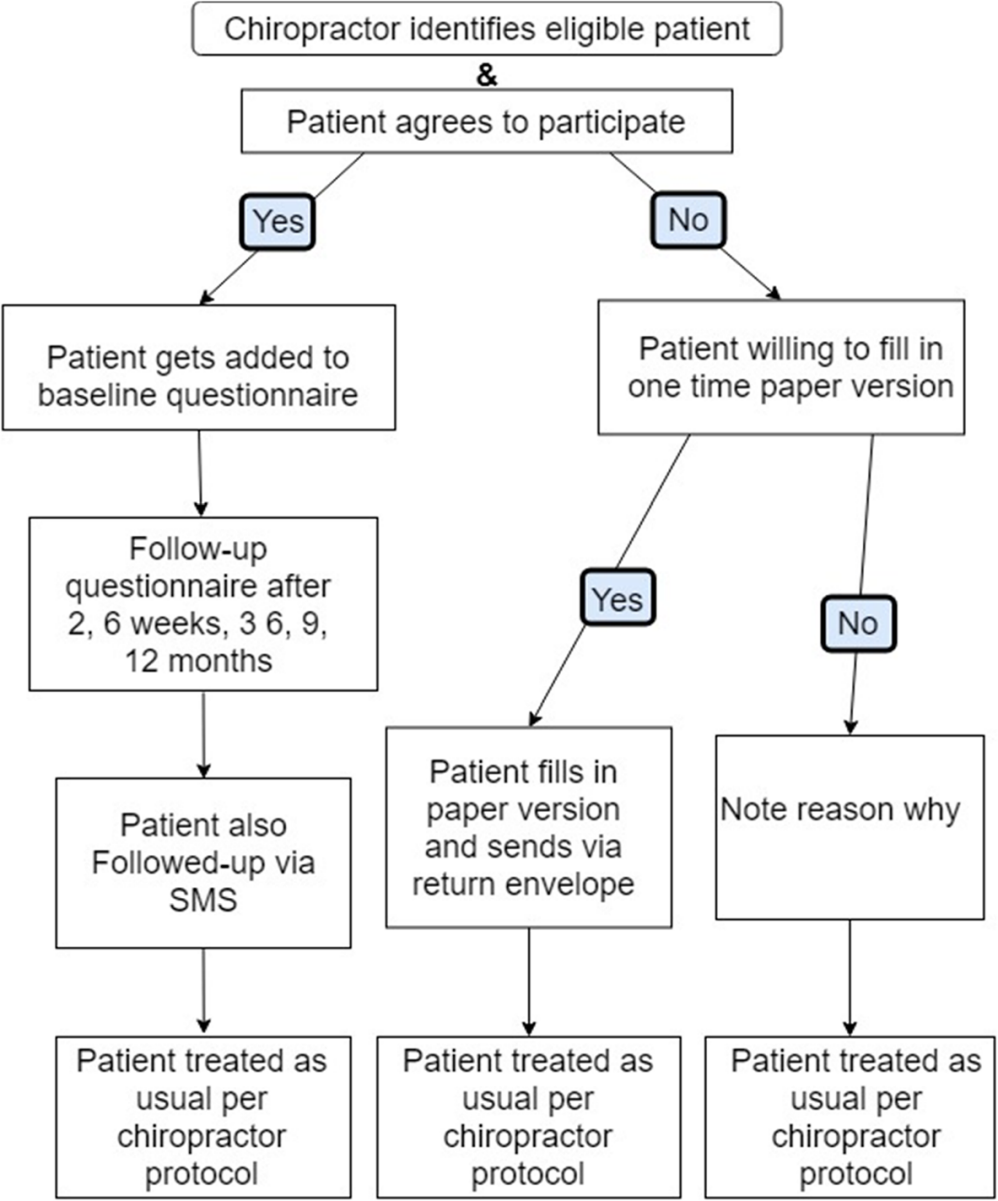
Flow Chart of BACE-C Study

Participating chiropractors will be asked to refer all potential participants who fulfill the inclusion criteria to the online questionnaire, preferably prior to the first appointment. Participants will be briefly informed about the study procedures over the phone when they call to make an appointment or during the initial consultation with the chiropractor. The chiropractor or chiropractic assistant will ask for the patient’s permission to send an email with a link to the informed consent and baseline questionnaire, so that it can be completed at home prior to the first visit or as soon as possible and no later than two weeks after the initial visit. Figure 1 shows the proposed flow of patient inclusion.

### Questionnaires

Links to the questionnaires will be sent by email and completed as a web-based questionnaire at baseline, 2 and 6 weeks, and at 3, 6, 9 and 12 months after the initial visit. In Sweden data will not be collected after 6 weeks because of logistical burden. Table 1 shows the measurements per follow-up round and the time frame for data collection.

**Table 1:** Content of the patient questionnaires

Demographics	Baseline	2 weeks	6 weeks	3 months	6 months	9 months	12 months
Age	X						
Gender	X						
Ethnicity	X						
Education level	X						
Marital Status	X						
Weight (for BMI)	X						
Height (for BMI)	X						
Primary Outcome Measures							
Global Perceived Effect		X	X	X	X	X	X
Recurrence of back pain		X	X	X	X	X	X
Severity of pain (11-point numeric rating scale)	X	X	X	X	X	X	X
Roland Morris Disability Questionnaire	X	X	X	X	X	X	X
E5-Q5-DL	X						
Cost Evaluation/ Healthcare Satisfaction	X	X	X	X	X	X	X
Adverse Events to Treatment		X	X	X	X	X	X
Pain Factors							
Duration, onset of symptoms, frequency, radiation, numbness, weakness	X						
Expectations of recovery	X						
Satisfaction with the current physical condition	X						
Lifestyle Factors							
Physical activity: International Physical Activity Questionnaire	X						
Smoking	X						
AUDIT-C Questionnaire	X						
Pittsburgh Sleep Quality Index	X						
Comorbidity Questionnaire	X						
Psychosocial Factors							
STarT Back Screening Tool	X						
Physical Exam							
Get Up & Go Test	X						

The primary outcome measures are: 1) low back pain intensity, 2) back-specific functional status and 3) global perceived effect. As a secondary measure, 4) healthcare costs will be measured. All outcomes are self-reported.

Patient-related factors: The following factors will be measured at baseline: 1) sociodemographic characteristics (i.e. age, gender, marital status, education level, height, weight); 2) physical activity (measured with the International Physical Activity questionnaire [15]); 3) other lifestyle variables smoking; measured by pack years, alcohol use measured by the short version of the AUDIT-C [17, 18], sleeping habits; measured by the short version of the Pittsburgh Sleep Quality Index [19]; 4) comorbidities using the Self-administered Comorbidity Questionnaire [20] and 7) indicator screening tool (STarT Back) for poor outcome [21, 22] and 5) quality-of-life measured with the EQ-5D-5 L at baseline only. In Sweden the EQ-5D-3 L will be used. The EQ-5D measures five dimensions: mobility, self-care, usual activities, pain/discomfort and anxiety/depression [23, 24].

In the Netherlands, each chiropractor will also perform at the first consult the “timed Up & Go” test [25]. The “timed Up & Go” test is composed of a variety of movements which are necessary for daily activities: walking, standing up, turning, stopping, and sitting down; and predictive of falls in the elderly [25]. In previous studies, this test showed associations with quality-of-life scores [26].

#### Pain

Pain intensity will first be measured using an 11-point numerical rating scale (NRS) [27] in which 0 represents ‘no pain ‘and 10 represents ‘the worst pain ever’. Second, several questions about the severity and reoccurrence of complaints will be asked at all follow-up measurements. Questions will be about average pain in the previous 24 h, previous week, and where applicable in the follow up questions, the previous three months. Pain trajectory diagrams will be graphically produced to describe the course of pain over one year (Table 1).

#### Back-specific functional status

Functional status will be measured at baseline and all follow-up intervals using the Roland Morris Disability Questionnaire (RMDQ) [28], in which total score can range from 0 (no disabilities) to 24 (severe disabilities).

#### Global perceived effect

Global perceived effect (GPE) will be measured on a 7-point scale, ranging from ‘completely recovered’ to ‘worse than ever’ [28, 29]. Patients will be asked to provide additional (open-ended) explanation if they report worse or much worse global perceived effect compared to the previous follow-up measurement. GPE will be dichotomized for the analyses as follows: ‘completely recovered’ and ‘much better’ will be considered ‘improved’, while all other responses will be considered ‘not improved’ [30].

#### Healthcare consumption

Healthcare consumption will include the use of all primary health care (e.g. general practitioner, physiotherapist), all secondary healthcare (e.g. hospital based neurologist, orthopedic surgeon), hospitalization, complementary care (e.g. acupuncture, dry needling, massage) as well as the use of both prescribed and over the counter medication. Questions were adapted based on the iMTA medical consumption questionnaire [31]. Healthcare consumption characteristics will be valued in accordance with costing guidelines of each participating country, such as the Dutch Manual of Costing [32].

#### Chiropractor-related factors

These variables will be obtained from the chiropractors themselves: 1) sociodemographic (age, gender), school attended (school, year of graduation), and types of treatments commonly delivered in their practice. Data collection is based upon consecutive inclusion of patients, but recognize that this may represent ‘convenience sampling’. Ideally, the patient is to complete the baseline questionnaire prior to the first appointment; however, it may be completed following the first visit. At the end of the data collection period, chiropractors will also be asked to submit treatment dates of patient during the year of inclusion, which will give an indication of dosage and frequency. These characteristics will be included where deemed relevant in the analysis.

In the Netherlands and in Sweden, each chiropractor will be asked to fill in several questions about their expectations of patient recovery. This will be asked at the first four treatment visits.

### Statistical analyses

#### Descriptive analyses

Baseline variables will be presented as percentages for categorical variables and as means plus standard deviations for continuous variables. In case of non-normal distributions, continuous variables will be described as medians with corresponding interquartile ranges. Furthermore, descriptive information of the primary and secondary outcome variables will be presented for baseline and all follow-up intervals. Descriptive analyses will be conducted for the entire data set from all participating countries as well as stratified for each country.

The primary objective will be answered using the entire data set from all participating countries and subsequently stratified by country.

In addition, the primary objective will be answered for each primary outcome separately by multilevel models with three levels (observations over time clustered within patients, clustered within practices). Country will be included as a covariate in the models (as dummy variables) [33]. The models will thus include time as a continuous variable as well as country as independent variables. Potential need for time squared and time cubed will be investigated by assessing the significance level of the quadratic and/or cubic terms. A random intercept will be included a priori. The need for a random slope for time will be investigated by the likelihood ratio test, in a stepwise manner [33].

The clinical course of pain and back-specific functional status will be analyzed by linear multilevel models, global perceived effect by logistic multilevel models and healthcare costs by a linear multilevel model with bootstrapped confidence intervals because of the expected skewed distribution of the cost data. We will report regression coefficients (linear models), odds ratios (logistic models), corresponding 95% confidence intervals and two-sided *p*-values.

Given that the baseline data collection allows for flexibility of inclusion (i.e. patients may complete prior to or following their first visit), this broad window for data collection has the potential to significantly impact baseline values, and subsequently each of the three primary outcome measures. Therefore, in order to deal with this problem, we will correct for this in the analyses, specifically the pain trajectories and reporting outcome. Patients are to be included consecutively, but we recognize that inclusion represents a ‘convenience sample’.

We know when patients completed the baseline questionnaire and will examine in a sensitivity analysis whether there are differences in the outcomes between those who complete the questionnaire prior to the first visit as opposed to completing if after their first visit. A paper version was designed for those who prefer not to participate, yet willing to complete a questionnaire one-time only prior to the first visit. We will compare both patient groups on demographic information as well as the primary outcomes in order to determine whether there is selection bias in our sampling. We recognize that those who receive ‘care-only’ or ‘care-only plus maintenance/supportive care’ may have different trajectories and outcomes; therefore, we will stratify these analyses for these groups should there appear to be fundamental differences. In order to classify this variable, two of the investigators (AJ, SMR) will examine the frequency and dosage of care for the entire year, independently of one another. We do not have a specific hypothesis that we are testing; therefore, we did not perform a power calculation. As this is an international study, we expect to be able to recruit approximately 600 patients in order to answer the primary objectives.

## Discussion

This study is to our knowledge the first large-scale, prospective, multicenter, international study to be conducted in a chiropractic setting and the first one focusing on older adults with low back pain consulting a chiropractor. The primary objectives of the BACE-C study are to examine the clinical course over one year of the intensity, healthcare costs and improvement rates of low back pain in patients aged 55 and older who consult a chiropractor for a new episode of low back pain, meaning low back pain for the first time or those patients who have not been to a chiropractor in the previous six months. This is independent of whether they have seen another type of health care provider for the current episode. By understanding the impacts of various factors on the course and treatment of low back pain in the elderly population, this large data set will allow us to provide input for the development of future feasibility intervention studies in this patient group.

We will not be linking our database with claims data as these are not readily available, and we do not have the resources to do this, but will rely on self-reported data from participating patients. We recognize that this is a potential limitation, because of possible information bias. However, we aim to estimate total healthcare consumption, including OTC and non-declared care as well (which is not necessarily available from claims data). As well baseline data will be collected at various time points either before or after patients has been treated. This could also be a possible limitation and the software will timestamp the questionnaires and we will also receive treatment dates from the chiropractors, so we can minimize this limitation and evaluate the baseline date as covariates in the analysis.

An additional limitation to the study is the timing of completion of the baseline questionnaire, where patients will be able to complete the questionnaire prior to or following the first visit. This flexibility was introduced in order to make patient inclusion for the chiropractors easier; however, it does introduce a potential limitation, namely, such a broad window for baseline data collection has the potential to impact the primary outcome measures. Our plan is to correct for potential differences in the analysis. The most important implication of this study is that it will provide data where these are currently lacking. Specifically, this study will give insight into the use and course of chiropractic care in the elderly with low back pain. This is consistent with the call to action from the recent Lancet series on low back pain. The results from this study may influence clinical practice if it means that the course, costs and improvement for this population are different than their younger counterparts with low-back pain. We invite other research groups worldwide to join the BACE-C consortium.

### Data management, storage and security

Data will be stored on institutional network drives with firewalls and security measures in place according to national and European Union data protection regulations. Hard copy records will be stored in a locked cabinet in a secure location. Access to records and data will be limited to study personnel. Study data will be de-identified and a master log file with identifiers will be kept and stored separately from the data. Only anonymized data will be used for analyses.

## Data Availability

Data sharing is not applicable to this article as no datasets were generated or analyzed during the current study.
